# Changes in actigraphy-assessed sleep from childhood to adolescence: The role of neighborhood socioeconomic disadvantage

**DOI:** 10.1016/j.sleh.2025.04.002

**Published:** 2025-05-19

**Authors:** Thomas E. Fuller-Rowell, Megan M. Zeringue, Ekjyot K. Saini, Samia Sultana, Mona El-Sheikh

**Affiliations:** a Department of Human Development and Family Science, Auburn University, Auburn, Alabama, United States; b Department of Psychology, Middle Tennessee State University, Murfreesboro, Tennessee, United States; c Department of Human Development and Family Studies, Pennsylvania State University, University Park, Pennsylvania, United States

**Keywords:** Neighborhood socioeconomic status, Neighborhood poverty, Longitudinal analyses, Sleep actigraphy, Racial disparities, Black/African American

## Abstract

**Objectives:**

Few studies have examined longitudinal effects of neighborhood socioeconomic disadvantage on objectively assessed sleep outcomes among youth. This study examined neighborhood disadvantage as a predictor of changes in actigraphy-assessed sleep over a 6-8-year period from childhood to adolescence. Racial/ethnic differences in effects were also considered.

**Methods:**

Participants were 339 children residing in small towns and semirural contexts within the Southeastern region of the United States (*M*_age T1_ = 10.3 years, *SD* = 0.8; 46% female; 36% Black, 64% White; *M*_age T2_ = 17.6 years, *SD* = 0.8). Sleep duration (from onset to wake time) and quality/continuity (efficiency and long wake episodes) were assessed using wrist actigraphy. Neighborhood disadvantage was assessed from established census tract measures geocoded to childhood residential addresses.

**Results:**

Sleep duration decreased between childhood and adolescence and sleep quality/continuity increased. Neighborhood disadvantage was associated with decreases in sleep quality/continuity from childhood to adolescence, but was not associated with sleep duration. Neighborhood effects remained significant after adjusting for family socioeconomic status. Interaction effects between neighborhood disadvantage and race indicated that the magnitude of neighborhood effects on changes in sleep quality/continuity were larger for Black youth than for White youth.

**Conclusions:**

This study is the first to show that objectively assessed neighborhood disadvantage is associated with longitudinal changes in actigraphy-assessed sleep quality from childhood to adolescence, and that this association was larger for Black youth than for White youth. The results suggest that neighborhood factors may be key to addressing widening racial disparities in sleep across this developmental period.

A growing scientific knowledgebase suggests that childhood and adolescence are key developmental periods for understanding sleep. Shorter and more disturbed sleep among youth have been consistently associated with poorer self-regulation,^1^ cognitive functioning,^2^ academic performance,^3^ and subsequent mental and physical health.^4,5^ With US children and adolescents getting significantly less than recommended amounts of sleep, addressing poor sleep is also increasingly recognized as a pressing public health priority,^6,7^ and identifying social determinants of changes in sleep from childhood to adolescence is of high scientific importance. Examining predictors of change by adjusting for baseline levels, helps to distinguish between between-person differences and within-person change and moves in the direction of establishing causal relationships.^8^ So doing provides much-needed information to inform public health interventions that seek to improve sleep and reduce sleep disparities during this critical developmental period.^9^

An expanding literature also suggests that neighborhood socioeconomic disadvantage is associated with shorter sleep duration and more disturbed sleep among children, adolescents, and adults.^10–13^ However, the degree to which objectively assessed neighborhood socioeconomic contexts are associated with longitudinal changes in sleep duration and quality from childhood to adolescence is not known. Furthermore, race differences in the effects of neighborhood socioeconomic status (SES) on sleep across this period are not well characterized. The significance of these knowledge gaps is underscored by two lines of research.

First, while evidence of a cross-sectional association between neighborhood SES and sleep is growing, longitudinal studies are scarce.^14,11,15^ Reviews of literature on children and adolescents have concluded that lack of objective sleep outcome assessment and use of primarily cross-sectional study designs were salient limitations of extant research on neighborhoods and sleep.^12^ Some of the data sources used to examine neighborhood effects on sleep have multiple within-person assessments.^12,16–18^ However, very few published studies have reported on neighborhood SES as a predictor of changes in sleep outcomes over time.

The current literature provides key evidence that examining longitudinal associations is warranted. For example, one important study modeled the cross-sectional association between neighborhood disadvantage and sleep at three time points and included person-level fixed effects.^19^ This approach allows for consideration of whether cross-sectional associations remain after accounting for unmeasured person-level confounders. The results showed that a portion of the association between neighborhood disadvantage on sleep outcomes remained. Other research has shown that neighborhood associations with sleep are evident across multiple developmental periods.^11^ However, many of the studies designated as longitudinal in reviews of the literature do not examine neighborhood SES as a predictor of changes in sleep, and thus little is currently known about the degree to which neighborhood SES influences changes in subsequent sleep quality or duration, particularly between the important developmental periods of childhood and adolescence. Studies examining the longitudinal association between neighborhood SES and sleep are thus sorely needed and of high significance in efforts to advance science on neighborhood factors as determinant of sleep.

Second, there is growing evidence that neighborhood factors play a key role in Black-White health disparities in the United States.^20–22^ The US Centers for Disease Control and Prevention and other government agencies have set priorities to improve neighborhood conditions as a strategy to address health disparities.^10,23^ Disparities in sleep duration and quality between Black and White youth are well documented,^24–28^ and recent evidence suggests that these disparities may widen across the period from childhood to adolescence.^29^ Understanding social determinants of sleep among racial/ethnic minority youth across this developmental period is therefore a high priority.

Neighborhood socioeconomic disadvantage may also play a more detrimental role for Black youth. Structural racism and a long history of discriminatory policies make it more likely for Black youth to grow up in poor neighborhoods and for poor neighborhoods with a greater proportion of racial/ethnic minority residents to be characterized by particularly high levels of adversity.^30,31^ This is true in both urban and rural contexts across the United States, including in the Southeastern settings from which the current sample was recruited.^14,21,32^ However, the role of neighborhood disadvantage as a predictor of sleep outcomes from childhood and adolescence among Black and White youth is not well understood. Based on these lines of research, our overarching hypotheses were that neighborhood socioeconomic disadvantage would be associated with decreases in sleep duration and quality from childhood to adolescence, and that the deleterious longitudinal consequences of neighborhood disadvantage would be greater for Black youth than White youth.

## Method

### Participants

Data for the current study come from multiple waves of a longitudinal investigation examining biopsychosocial influences on health in the family (Auburn University Sleep Study; data collected between 2010 and 2020). Families were recruited from two public school districts in the Southeastern United States through letters sent home with children; families who were interested in participating were asked to call the investigators to schedule lab visits and sleep assessments. To qualify for inclusion in the study, parents did not report a diagnosed learning disability or sleep disorder for the target child (see^33^ for further detail about initial recruitment).

Following initial participation, families were asked to complete assessments again at two additional waves in childhood, each 1 year apart, and a fourth wave in adolescence, 6-8 years later (*M* = 6.7 years, *SD* = 0.55). Of the 339 youth who participated in the childhood assessments (T1), 167 also participated in the adolescent assessment (T2). See analysis plan section for a discussion of missing data. The final analytic sample included 339 youth who lived across 41 census tract neighborhoods in childhood (53.6% male, 46.4% female; 63.9% White, 36.1% Black). The sample mean age was 10.30 years at the childhood assessment (*SD* = 0.76) and 17.65 years at the adolescent assessment (*SD* = 0.78). Childhood census tract was coded at the first available time point at which families participated (81.7% at age 10 assessment, the remainder at age 11). All other childhood variables (i.e., family SES) were averaged across the three childhood waves (ages 10, 11, and 12).

### Procedure

The study was approved by the university’s Institutional Review Board. Parents provided written consent for their child’s participation, and written assent was also obtained during adolescence. At each wave of data collection, actigraphs were delivered to the families’ homes or provided to them during a campus lab visit, and the youth were asked to wear them for seven consecutive nights. Youth received nightly reminders to wear the actigraph and to fill out a nightly sleep log.

### Measures

#### Childhood neighborhood socioeconomic disadvantage

Neighborhood characteristics were geocoded by linking participants’ home addresses at their first year of participation in the study to tract-level data from the 2012 American Community Survey. Consistent with prior work,^14,21,34^ an aggregate index of neighborhood socioeconomic disadvantage was scored as the mean of five standardized variables: percent in poverty, percent with less than a high school education, median household income (reverse coded), percent with a college degree or greater (reverse scored), and proportion of households receiving public assistance (α = 0.81). Participants were distributed across 41 census tracts in Alabama and neighboring regions of the Southeastern United States.

#### Sleep

At each time point, youth wore Octagonal Basic Motionlogger actigraphs (Ambulatory Monitoring, Ardsley, NY) on their non-dominant wrist at home while sleeping for seven consecutive nights. Data were scored in 1-minute epochs using zero-crossing mode with the Action W2 software, and sleep was scored using the Sadeh algorithm.^35^ Sleep onset and wake times were derived from the actigraph and corroborated with the youth’s sleep log. Sleep onset was determined from the first three consecutive minutes scored as sleep after self-reported bedtime, and wake time was determined from the last five consecutive minutes scored as sleep before reported wake time. Sleep data for participants with fewer than five nights were treated as missing. Sensitivity analyses showing results for three nights instead of five as an alternative inclusion criteria are shown in the online Appendix, Tables A1–A3.

Sleep parameters were defined in accordance with the terminology used in the manual for the actigraph and its associated software (Ambulatory Monitoring Inc, Ardsley, NY). *Sleep duration* was derived as the number of minutes scored as sleep between onset and wake time. *Sleep efficiency* was derived as the percentage of epochs scored as sleep between sleep onset and wake time. In some prior work, this index has been referred to as percent sleep.^36^ *Long wake episodes* were derived as the number of wake episodes lasting 5 minutes or longer between onset and wake time. Long wake episodes was selected as a measure of sleep quality because it is an established measure in youth samples,^37–39^ is an excellent indicator of insomnia,^40^ and captures a distinct component of sleep (less highly correlated with efficiency than alternative indicators). Sleep parameters were first averaged across all available nights at each time point. Indices at waves 1, 2, and 3 were significantly correlated for sleep duration (*r*s = 0.36-0.40), efficiency (*r*s = 0.29-0.38), and long wake episodes (*r*s = 0.39-0.47), all *p*s < .001, and were averaged to form composites of childhood sleep duration, efficiency, and long wake episodes. Measures of sleep timing (onset time, sleep midpoint) were also considered in supplemental analyses but yielded no significant results. These findings are shown in the online Appendix (Tables A4 and A5).

#### Covariates

At each time point, parents identified their children’s gender, race, and age, as well as their family’s yearly income, the number of individuals living in the household, and the highest level of education completed by each parent. Child age was averaged across the three childhood waves. Income-to-needs ratio (INR) was calculated at each wave by dividing family income by the federal poverty threshold for family size in the respective year (US Department of Commerce; http://www.commerce.gov/); the average INR across the three childhood waves was computed for use in analyses. Approximately 32.4% of families were living in poverty (INR < 1), 27.1% were near the poverty line (1 < INR ≤ 2), 22.9% were considered lower middle class (2 < INR ≤ 3), 12.2% were middle class (3 < INR ≤ 4), and 5.4% were upper middle class (INR > 4). Furthermore, the highest level of education completed by either parent at the first available time point was used as an additional covariate indexing SES. Most parents were high school graduates (24.8%) or attended some college without obtaining a degree (44.1%). Relatively few parents obtained less than a high school degree (4.8%), standard college degree (17.3%), or a graduate degree (9.0%).

### Analyses and missing data

A series of multilevel models were fit using Mplus V8.4 to account for clustering of individuals (level 1) within census tracts (level 2). For each sleep parameter, three separate models were fit. Model 1 examined neighborhood economic disadvantage as a level 2 predictor of adolescent sleep while controlling for child sleep, age, gender, and race as level 1 predictors. Model 2 added family INR and parent education to ascertain the unique effects of neighborhood disadvantage beyond family SES. Model 3 then considered the cross-level interaction between neighborhood disadvantage and race to examine potential race difference in neighborhood effects. Fitted interaction plots were created for statistically significant interactions. Specifically, the magnitude of changes in sleep parameters from childhood to adolescence was plotted for high and low ( ± 1 *SD*) levels of neighborhood disadvantage for Black and White youth. The results reported in the tables and figures are unstandardized, and standardized effect sizes are reported in the text. Standardized effect sizes can be interpreted as magnitudes of change in standard deviation units (defined by a standard deviation at the baseline assessment). Missing data were handled using full-information maximum likelihood estimation. Of the 339 individuals included in analyses, 13% had missing data for family income, 6% on T1 sleep measures, and 3% for neighborhood disadvantage. At the T2 assessment (6-8 years later), 51% had missing sleep data. Missing data at the adolescent follow-up assessment were due to attrition (199 out of 339 participated in the follow-up and 167 of 199 had valid actigraphy data). Reasons for attrition included no longer residing in the area or nonresponse to contacts for follow-up participation (e.g., due to changes in contact information). Missingness of data at the follow-up was not significantly correlated with neighborhood SES or sleep outcomes but showed a small correlation with family income (*r* = 0.183, *p* = .002) and parent education (*r* = .196, *p* < .001). Descriptive statistics for the subsample who participated at both time points is provided in the Appendix (Table A6). Family SES variables are included as covariates in our models and thus bias due to missing data in the reported results is expected to be minimal. To further probe this assumption, additional analyses were conducted to examine whether the reported results differed when only those with sleep data at T2 were included in the analyses. The general pattern of findings and significance was equivalent to those reported in the primary analyses (see online Appendix, Tables A7–A8).

## Results

Descriptive statistics for sleep variables are shown in Table 1. Independent-sample *t*-tests revealed that families of Black children had lower income-to-needs and lived in more disadvantaged neighborhoods, on average, compared with families of White children. Additionally, Black youth experienced significantly shorter sleep duration than White youth in both childhood and adolescence. There were no significant race differences in sleep efficiency or long wake episodes in childhood. However, Black youth had significantly lower sleep efficiency and more long wake episodes than White youth during adolescence. Repeated-measures *t*-tests revealed that participants’ sleep was significantly shorter, *t*(163) = 7.41, *p* < .001, more efficient, *t*(163) = −10.08, *p* < .001, and contained fewer long wake episodes, *t*(163) = 11.38, *p* < .001, in adolescence compared with childhood.

Compared with girls, boys experienced shorter sleep duration, *t*(316) = 3.02, *p* = .003, and lower sleep efficiency, *t*(316) = 2.19, *p* = .029, in childhood, as well as shorter sleep duration in adolescence, *t*(165) = 2.86, *p* = .005. There were no significant gender differences in adolescent sleep efficiency, childhood or adolescent long wake episodes, or family INR.

### Neighborhood disadvantage as a predictor of changes in sleep efficiency

Model results for predictors of sleep efficiency in adolescence are shown in Table 2. In model 1, childhood neighborhood economic disadvantage was associated with smaller increases in sleep efficiency from childhood to adolescence (*p* = .002). Specifically, youth living in neighborhoods with higher neighborhood disadvantage (1 *SD* above the mean) experienced a 0.56 *SD* increase in sleep efficiency in adolescence, whereas those living in neighborhoods with lower disadvantage (1 *SD* below the mean) experienced a 0.90 *SD* increase in sleep efficiency. Race was also associated with the magnitude of change in sleep efficiency (*p* = .007); Black youth experienced a 0.72 *SD* increase in sleep efficiency, whereas White youth experienced a 1.11 *SD* increase.

In model 2, INR and parent education were added as controls to determine whether neighborhood economic disadvantage continued to predict sleep efficiency beyond the effects of family SES. A higher INR was significantly associated with higher sleep efficiency (*p* = .03), while parent education did not significantly predict adolescent sleep efficiency. Childhood neighborhood disadvantage remained a significant predictor (*p* = .004).

**Fig. 1.**
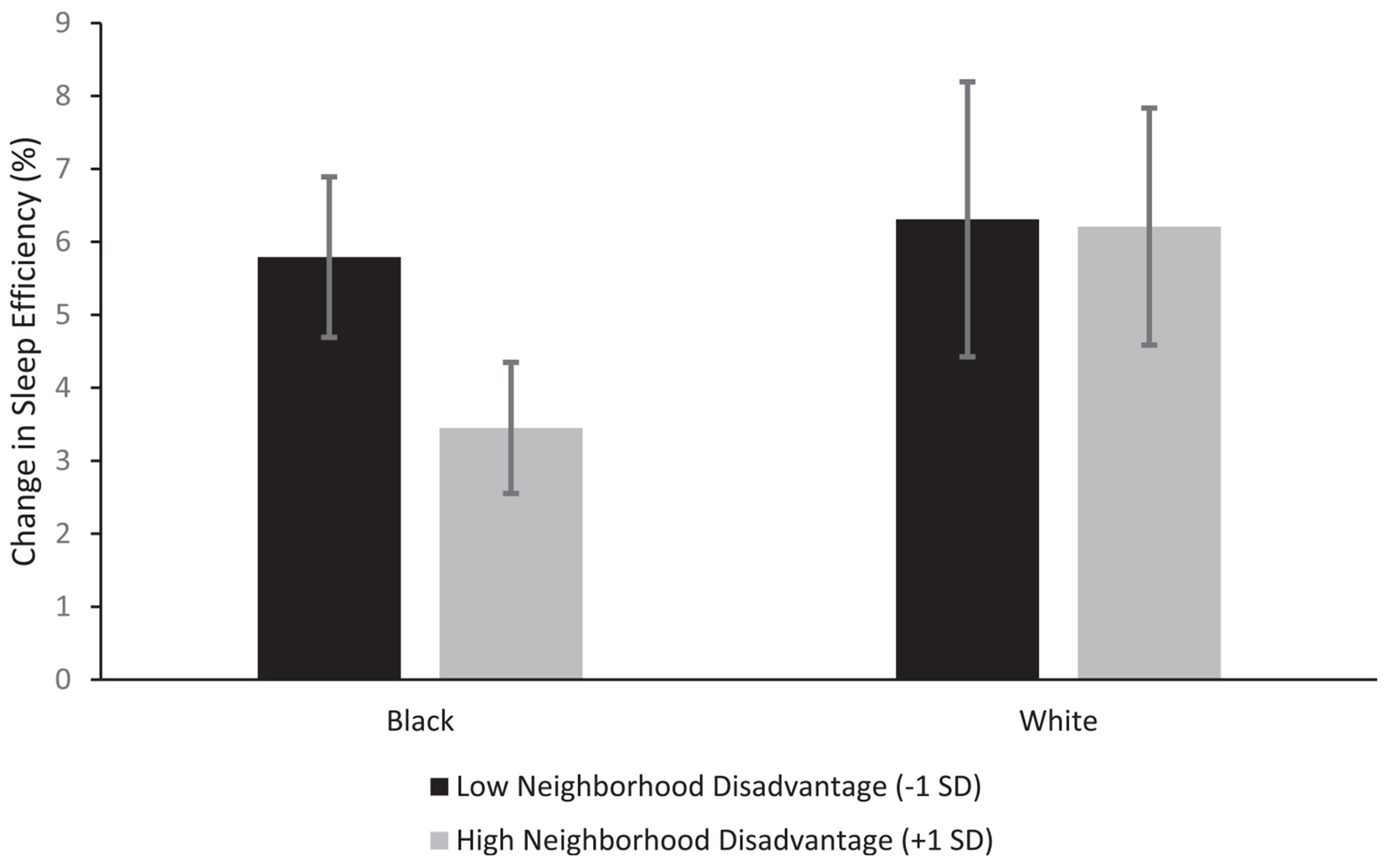
Magnitude of change in sleep efficiency from childhood to adolescence for Black (*n* = 122) and White (*n* = 216) participants at low (−1 *SD*) and high (+1 *SD*) levels of neighborhood socioeconomic disadvantage. Error bars represent ± 1.96 standard errors

Model 3 examined whether the association between neighborhood disadvantage and sleep efficiency varied as a function of race. The Neighborhood Disadvantage x Race interaction was a marginally significant predictor of sleep efficiency (*p* = .059). Notably, when family SES was excluded from the model, the Neighborhood Disadvantage x Race interaction term was significant (*B* = 1.435, *SE* = 0.493, *p* = .004). The moderation effect indicated that the sleep efficiency of Black children living in neighborhoods with high levels of economic disadvantage increased significantly less than that of Black children from less disadvantaged neighborhoods (Fig. 1). Specifically, Black children living in neighborhoods with higher economic disadvantage (+1 *SD*) experienced a 0.56 *SD* increase in sleep efficiency in adolescence, whereas Black children in neighborhoods with lower disadvantage (−1 *SD*) experienced a 1.06 *SD* increase in sleep efficiency. White children experienced the largest increases in sleep efficiency, regardless of neighborhood disadvantage (1.07 *SD* increase in efficiency in more disadvantaged neighborhoods and 1.09 *SD* increase in less disadvantaged neighborhoods).

### Neighborhood disadvantage as a predictor of changes in long wake episodes

Model results showing predictors of long wake episodes in adolescence are shown in Table 3. In model 1, childhood neighborhood economic disadvantage was associated with smaller decreases in long wake episodes from childhood to adolescence (*p* = .010). Youth living in neighborhoods with higher economic disadvantage (+1 *SD*) experienced a 0.68 *SD* decrease in long wake episodes in adolescence, whereas those living in neighborhoods with lower disadvantage (−1 *SD*) experienced a 0.86 *SD* decrease in long wake episodes. Additionally, on average, Black youth experienced a 0.77 *SD* decrease in long wake episodes in adolescence, whereas White youth experienced a 0.95 *SD* decrease in long wake episodes (*p* = .001).

In model 2, the inclusion of family SES variables indicated that neither INR nor parent education was associated with long wake episodes, and childhood neighborhood disadvantage remained a significant predictor (*p* = .021).

**Fig. 2.**
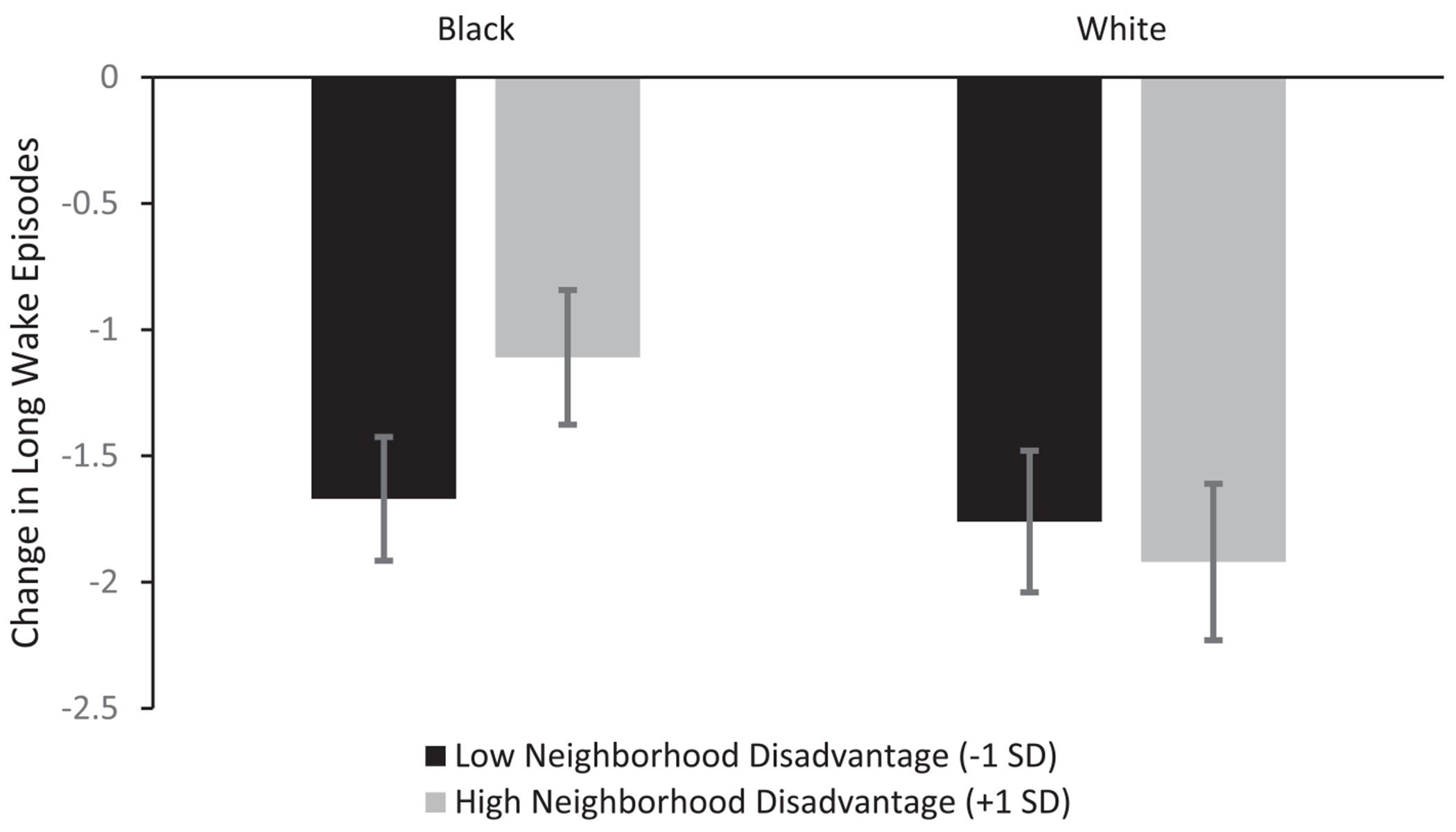
Magnitude of change in long wake episodes from childhood to adolescence for Black (*n* = 122) and White (*n* = 216) participants at low (−1 *SD*) and high (+1 *SD*) levels of neighborhood socioeconomic disadvantage. Error bars represent ± 1.96 standard errors

In model 3, the Neighborhood Disadvantage x Race interaction was a significant predictor of long wake episodes (*p* = .001). The moderation effect indicated that the decrease in long wake episodes was significantly less for Black children living in neighborhoods with high levels of economic disadvantage compared with Black children from less disadvantaged neighborhoods (Fig. 2). Specifically, Black children living in neighborhoods with higher economic disadvantage experienced a 0.59 *SD* decrease in long wake episodes in adolescence, whereas Black children in neighborhoods with lower disadvantage experienced a 0.99 *SD* decrease in long wake episodes. White children experienced similar declines in long wake episodes, regardless of neighborhood disadvantage (0.98 *SD* decrease in less disadvantaged neighborhoods and 1.08 *SD* decrease in more disadvantaged neighborhoods).

### Neighborhood disadvantage as a predictor of changes in sleep duration

Model results showing predictors of sleep duration in adolescence are reported in the online Appendix (Table A9). Across all models, childhood neighborhood economic disadvantage was not a significant predictor of sleep duration. White youth and females experienced longer sleep duration compared with Black youth and males, respectively. Additionally, race did not moderate the association between neighborhood disadvantage and sleep duration.

## Discussion

Adequate sleep duration and quality are essential for the healthy development of all youth,^41^ and are central mechanisms by which social stress gets under the skin to influence physiologic dysregulation and biological weathering.^42,43^ Understanding how neighborhood contexts influence sleep in childhood and adolescence will inform efforts to improve mental and physical health and address group disparities. The current study is among the first to examine neighborhood socioeconomic disadvantage in childhood as a predictor of longitudinal changes in actigraphy-assessed sleep between childhood and adolescence. The results indicate that neighborhood SES in childhood was associated with changes in actigraphy-assessed sleep quality between childhood and adolescence. Children residing in less advantaged neighborhoods had smaller improvements in sleep efficiency and long wake episodes than their more advantaged peers. Neighborhood SES was not a significant predictor of changes in sleep duration.

These findings are consistent with prior research showing that neighborhood socioeconomic disadvantage is associated with actigraphy-assessed sleep quality but not duration or timing^14,34^ and extend this work by showing that these associations held in longitudinal analyses. Longitudinal effects of neighborhood SES on sleep outcomes also remained significant after adjusting for family SES. The results build on important work showing that other characteristics of neighborhoods, such as crime, violence, or safety concerns, are also associated with adolescent sleep outcomes.^44–46^ Overall, this growing body of research brings attention to the neighborhood context as a consequential predictor of sleep health. In the current study, we showed that the magnitude of change in sleep outcomes from childhood to adolescence was significantly less positive/salubrious among children living in disadvantaged neighborhoods. Effect size estimates suggest that the difference in the magnitude of change in sleep quality between those living in advantaged and disadvantaged neighborhoods, was approximately a third of a *SD* for sleep efficiency (0.34) and a fifth of a *SD* different for long wake episodes (0.20). This difference is on par with the effect sizes for the influence of regular physical activity on sleep efficiency in meta-analyses.^47^

The reported null findings relating to the longitudinal association between neighborhood SES and actigraphy-assessed sleep duration were not expected in light of existing research showing that lower neighborhood SES is often correlated with shorter sleep duration.^12^ Nonetheless, the results are consistent with research showing that neighborhood disadvantage is often more strongly associated with measures of sleep quality than duration in both adolescent and adult samples.^21,48^ The reasons for nonsignificant effects on sleep duration are not clear, but could relate to the longitudinal study design, which focuses our analyses on change in sleep over time, or the specifics of the study sample (discussed further below). Another possibility is that declines in sleep duration from childhood to adolescence are normative, pervasive, and substantial and may overwhelm the effects of neighborhood social determinants in this context.^49^ Additional studies are needed to clarify the relative magnitude of neighborhood effects on specific sleep parameters at different points in the lifespan.

Another key finding was that the associations between neighborhood disadvantage and sleep quality were stronger for Black youth than for White youth. This finding was similar for sleep efficiency and long wake episodes. Two possible reasons for these differences should be noted. One is that neighborhoods with more Black residents may be more disadvantaged on a variety of characteristics due to the long history of segregation and structural racism that has undermined investment in Black communities.^50,32,51^ Another possibility is that Black youth may have fewer protective resources in their environment that could buffer the effects of adverse neighborhood conditions on health outcomes. For example, White youth in disadvantaged neighborhoods may have more access to extracurricular activities outside of their neighborhood due to differences in family resources and wealth.^52,53^ Future research will be needed to compare the relative contribution of these two possibilities, among others, and integrate them within broader conceptual frameworks on the environmental determinants of sleep.^54,55^ The findings of this research combine with previous studies to suggest that neighborhood disadvantage plays an important role in sleep disparities, and that policies that seek to address segregation and race differences in neighborhood risk factors (e.g., alleviation of restrictive zoning laws) have promising potential to mitigate these disparities. We also note that some prior research has reported that neighborhood disadvantage is associated with sleep duration among White but not Black youth.^56^ However, this work is based on self-reported bedtime and wake time at a single time point rather than objective or actigraphy assessments of duration and focuses on a later age range. The results are therefore not directly contradictory to those reported herein.

## Limitations

While our results are an important extension of prior work, several limitations are noteworthy. First, the findings should be interpreted in the context of the study design. The sample was recruited from semirural communities and small towns in Alabama. The results may therefore not generalize to other populations, such as those in urban areas. Some studies examining neighborhood disadvantage on health outcomes across rural and urban areas have found that neighborhood factors are a stronger predictor in rural than urban contexts.^57^ However, with respect to the association between neighborhood SES and sleep quality, the results of prior cross-sectional research in predominantly urban or suburban contexts are consistent with the current study.^21^ Furthermore, while some of the mechanisms for neighborhood effects on sleep may differ in urban and rural contexts, neighborhood disadvantage is likely to play an important role in both.^10^ Also noteworthy are findings in the literature suggesting that the prevalence of short sleep duration may be greater in urban than rural contexts.^58^ More research will therefore be important to examine whether neighborhood effects on sleep duration may be more pronounced in urban contexts.

Second, while our longitudinal study design and prospective analyses build importantly on prior research, our study design is observational and thus does not rule out reverse causality or the effects of confounding variables that might influence both neighborhoods of residence and changes in sleep. Parent characteristics, food security, or measures of psychosocial stress variables are examples of possible confounders or mediating mechanisms that could be considered in future research. Studies with larger sample sizes and more assessment time points will be useful to consider how changes in neighborhoods may impact changes in sleep outcomes. Instrumental variable analyses may also be fruitful to address the role of potential confounders. Examining changes in neighborhoods, however, comes with various methodological challenges that the field will need to more directly address in order to optimize scientific progress.

A third caveat relates to our assessment of neighborhood SES. Although our census indicators of neighborhood disadvantage are well established and objectively assessed, tracts do not always map well onto individuals’ lived experiences of their neighborhood context. Other types of objective neighborhood data such as those derived from different geographic levels (e.g., block group) or direct coding of neighborhood characteristics from street view images may help to supplement tract-based indicators. Self-report measures are also important to consider alongside objective indicators. Triangulation across multiple measurement approaches will likely be optimal in creating a more nuanced and accurate assessment of neighborhood influences.

Despite these limitations, the findings indicate that neighborhood socioeconomic disadvantage may play an important role in shaping changes in sleep quality from childhood to adolescence, particularly among Black youth. Addressing neighborhood disadvantage or supporting youth and families in these contexts through community investments or safety net policies will likely be important to improve sleep at the population level and address health disparities.

## Supplementary Material

Supplementary Materials

## Figures and Tables

**Table 1:** Descriptive statistics for study variables by race

	Full sample (*n* = 339)	Black (*n* = 122)	White (*n* = 216)	*p*
Childhood income-to-needs ratio	1.84 ± 1.23	1.23 ± 1.09	2.18 ± 1.17	< .001
Childhood neighborhood disadvantage	0.0002 ± 0.71	0.19 ± 0.86	−0.11 ± 0.59	.063
Childhood sleep duration (min)	448.84 ± 43.06	441.93 ± 40.41	452.65 ± 44.08	.033
Childhood sleep efficiency	88.79 ± 5.81	89.05 ± 5.68	88.64 ± 5.89	.548
Childhood long wake episodes	3.35 ± 1.78	3.19 ± 1.78	3.44 ± 1.78	.241
Adolescent sleep duration (min)	413.04 ± 62.99	394.05 ± 56.43	422.62 ± 64.18	.005
Adolescent sleep efficiency	94.13 ± 6.18	92.06 ± 7.03	95.17 ± 5.44	.002
Adolescent long wake episodes	1.72 ± 1.38	2.16 ± 1.54	1.50 ± 1.24	.004

Note. *p*-values for race differences are calculated using independent samples *t*-tests for person-level variables. To account for clustering of individuals within neighborhoods, multilevel regression models with race as the predictor were used to calculate *p*-values for race differences in neighborhood-level variables. The percentage of missing data on individual measures is reported in the “Methods” section. One participant has missing data for race.

**Table 2:** Model results for sleep efficiency in adolescence

Adolescent sleep efficiency	Model 1		Model 2		Model 3	
	B	SE	B	SE	B	SE
Intercept	4.16***	0.50	4.39***	0.43	4.62***	0.44
Sleep efficiency (T1)	0.42***	0.08	0.42***	0.07	0.41***	0.08
Age	0.41	0.33	0.38	0.34	0.36	0.33
Gender (male)	−0.72	0.75	−0.96	0.70	−0.86	0.71
Race (White)	2.27**	0.84	1.91*	0.81	1.64*	0.79
Income-to-needs			0.70*	0.32	0.71^+^	0.37
Parent education			0.11	0.39	0.14	0.40
Neigh. disadvantage	−0.79**	0.25	−0.67**	0.23	−1.17***	0.27
Neigh. disadvantage*Race					1.12^+^	0.59

Note. Unstandardized estimates are shown. All continuous predictor variables are grand mean-centered and *z*-scored to have a mean of zero and a standard deviation of 1 (*n* = 339).

+ *p* < .10,

* *p* < .05,

** *p* < .01,

*** *p* < .001.

**Table 3:** Model results for long wake episodes in adolescence

Adolescent long wake episodes	Model 1		Model 2		Model 3	
	B	SE	B	SE	B	SE
Intercept	−1.37***	0.12	−1.41***	0.10	−1.39***	0.11
Long wake episodes (T1)	0.31***	0.05	0.30***	0.05	0.29***	0.05
Age	−0.10	0.11	−0.09	0.11	−0.09	0.11
Gender (male)	0.19	0.15	0.24	0.14	0.16	0.15
Race (White)	−0.52**	0.16	−0.44**	0.16	−0.45**	0.14
Income-to-needs			−0.11	0.09	−0.10	0.10
Parent education			−0.09	0.08	−0.10	0.08
Neigh. disadvantage	0.13*	0.05	0.10*	0.04	0.28***	0.07
Neigh. disadvantage*Race					−0.36***	0.10

Note. Unstandardized estimates are shown. All continuous predictor variables are grand mean-centered and *z*-scored to have a mean of zero and a standard deviation of 1 (*n* = 339).

* *p* < .05,

** *p* < .01,

*** *p* < .001.

## Data Availability

Data will be made available after completion of the study in accordance with National Institutes of Health data sharing guidelines.

## Appendix A. Supporting information

Supplementary data associated with this article can be found in the online version at doi:10.1016/j.sleh.2025.04.002.
