# Supplementary material for: Changes in actigraphy-assessed sleep from childhood to adolescence: The role of neighborhood socioeconomic disadvantage
Source: Sleep Health. Author manuscript; Available in PMC 2025 Nov 12. (PMC12609179; doi:10.1016/j.sleh.2025.04.002)
Supplement: Supplementary Materials [file NIHMS2121977-supplement-Supplementary_Materials.pdf]

Appendix: Supplemental Materials

Table A1

Model Results for Sleep Duration in Adolescence (3 nights or more of actigraphy data instead of 5 or more)

Adolescent Sleep Duration	Model 1		Model 2		Model 3	
	B	SE	B	SE	B	SE
Intercept	-42.078***	7.55	-39.99***	7.29	-39.85***	8.02
Sleep Duration (T1)	0.43***	0.12	0.43***	0.12	0.42***	0.12
Age	6.73^{+}	3.83	6.28^{+}	3.80	4.23	3.78
Gender (Male)	-19.25*	8.27	-20.53*	8.47	-18.71^{+}	10.78
Race (White)	24.58**	8.84	21.56*	9.22	20.53	18.42
Income to Needs			7.71	5.61	7.29	5.98
Parent Education			-4.75	3.09	-3.67	4.48
Neigh. Deprivation	2.81	2.57	3.49	2.61	-0.50	3.21
Neigh. Deprivation*Race					8.54^{+}	5.13

Note. Unstandardized estimates are shown. All continuous predictor variables are grand mean centered and z-scored to have a mean of zero and a standard deviation of 1 (n = 339).
+$p < 0.10$. *$p < 0.05$. **$p < 0.01$. ***$p < 0.001$.

Table A2

Model Results for Sleep Efficiency in Adolescence (3 nights or more of actigraphy data instead of 5 or more)

Adolescent Sleep Efficiency	Model 1		Model 2		Model 3	
	B	SE	B	SE	B	SE
Intercept	4.47***	0.60	4.70***	0.48	4.99***	0.46
Sleep Efficiency (T1)	0.33***	0.07	0.31***	0.07	0.31***	0.07
Age	-0.04	0.40	-0.05	0.37	-0.03	0.43
Gender (Male)	-0.65	0.80	-0.89	0.66	-0.86	0.71
Race (White)	2.22*	0.90	1.91**	0.82	1.68^{+}	0.91
Income to Needs			0.45	0.36	0.46	0.40
Parent Education			0.39	0.29	0.37	0.42
Neigh. Deprivation	-0.75*	0.31	-0.66*	0.27	-1.16*	0.49
Neigh. Deprivation*Race					1.12	0.84

Note. Unstandardized estimates are shown. All continuous predictor variables are grand mean centered and z-scored to have a mean of zero and a standard deviation of 1 (n = 339).
+p < 0.10. *p < 0.05. **p < 0.01. ***p < 0.001.

Table A3

Model Results for Long Wake Episodes in Adolescence (3 nights or more of actigraphy data instead of 5 or more)

Adolescent Long Wake Episodes	Model 1		Model 2		Model 3	
	B	SE	B	SE	B	SE
Intercept	-1.40***	0.12	-1.45***	0.13	-1.42***	0.13
Long Wake Episodes (T1)	0.29***	0.05	0.28***	0.05	0.27***	0.05
Age	-0.04	0.11	-0.04	0.12	-0.03	0.13
Gender (Male)	0.15	0.13	0.21^{+}	0.13	0.14	0.14
Race (White)	-0.49**	0.19	-0.43*	0.19	-0.45**	0.17
Income to Needs			-0.06	0.10	-0.04	0.11
Parent Education			-0.15*	0.07	-0.16*	0.07
Neigh. Deprivation	0.14*	0.06	0.12*	0.05	0.31***	0.08
Neigh. Deprivation*Race					-0.39***	0.11

Note. Unstandardized estimates are shown. All continuous predictor variables are grand mean centered and z-scored to have a mean of zero and a standard deviation of 1 ($n = 339$).
*$p < 0.05$. **$p < 0.01$. ***$p < 0.001$.

Table A4

Model Results for Sleep Onset Time in Adolescence

Adolescent Sleep Onset Time	Model 1		Model 2		Model 3	
	B	SE	B	SE	B	SE
Intercept	112.91***	9.03	111.99***	9.42	112.78***	9.50
Sleep Onset Time(T1)	0.85***	0.12	0.86***	0.11	0.85***	0.11
Age	-5.97	5.56	-5.87	5.71	-5.50	5.99
Gender (Male)	-2.04	10.79	-0.844	10.59	-1.895	13.11
Race (White)	-1.187	10.33	1.05	10.05	0.00	9.86
Income to Needs			-3.136	7.20	-2.963	7.15
Parent Education			-3.348	6.95	-3.355	6.94
Neigh. Deprivation	-6.428	4.20	-7.23^{+}	3.89	-7.086	6.98
Neigh. Deprivation*Race					-0.024	12.55

Note. Unstandardized estimates are shown. All continuous predictor variables are grand mean centered and z-scored to have a mean of zero and a standard deviation of 1 (n = 339).
+p < 0.10. *p < 0.05. **p < 0.01. ***p < 0.001.

Table A5

Model Results for Sleep Midpoint in Adolescence

Adolescent Sleep Midpoint	Model 1		Model 2		Model 3	
	B	SE	B	SE	B	SE
Intercept	82.02***	9.11	81.99***	9.47	84.75***	9.60
Sleep Mid (T1)	0.87***	0.16	0.90***	0.16	0.91***	0.19
Age	-1.69	4.50	-1.82	4.57	-1.61	4.78
Gender (Male)	-10.68	9.63	-10.06	9.20	-11.07	11.63
Race (White)	1.93	10.19	3.81	10.00	0.081	9.95
Income to Needs			-0.14	5.47	0.26	6.37
Parent Education			-7.73	6.60	-7.83	6.65
Neigh. Deprivation	-3.582	4.04	-4.128	3.82	-4.31	13.29
Neigh. Deprivation*Race					-0.02	22.25

Note. Unstandardized estimates are shown. All continuous predictor variables are grand mean centered and z-scored to have a mean of zero and a standard deviation of 1 (n = 339).
+$p < 0.10$. *$p < 0.05$. **$p < 0.01$. ***$p < 0.001$.

Table A6

Descriptive Statistics for Study Variables by Sample

	Full Sample (n = 339)	Sample with data at the adolescent follow-up (n = 199)
Childhood Income to Needs Ratio	1.84 ± 1.23	1.99 ± 1.29
Childhood Neighborhood Disadvantage	.0002 ± .71	-0.01 ± .71
Childhood Sleep Duration (minutes)	448.84 ± 43.06	449.91 ± 43.83
Childhood Sleep Efficiency	88.79 ± 5.81	88.98 ± 6.01
Childhood Long Wake Episodes	3.35 ± 1.78	3.26 ± 1.72

Note. The percentage of missing data on individual measures is reported in the methods section.

Table A7

Model Results for Sleep Efficiency in Adolescence (with sample restricted to include only those with sleep data at the adolescent follow-up)

Adolescent Sleep Efficiency	Model 1		Model 2		Model 3	
	B	SE	B	SE	B	SE
Intercept	4.13***	0.50	4.37***	0.43	4.65***	0.44
Sleep Efficiency T1	0.42***	0.08	0.42***	0.07	0.41***	0.08
Age	0.42	0.33	0.37	0.34	0.36	0.33
Gender (Male)	-0.70	0.75	-0.97	0.70	-0.89	0.71
Race (White)	2.29**	0.84	1.92*	0.81	1.61*	0.79
Income to Needs			0.73*	0.32	0.74*	0.37
Parent Education			0.10	0.39	0.13	0.40
Neigh. Disadvantage	-0.77**	0.25	-0.64**	0.23	-1.15***	0.27
Neigh. Disadvantage*Race					1.10^{+}	0.59

Note.Unstandardized estimates are shown. All continuous predictor variables are grand mean centered and z-scored to have a mean of zero and a standard deviation of 1 (n = 339). +p < 0.10. *p < 0.05. **p < 0.01. ***p < 0.001.

Table A8

Model Results for Long Wake Episodes in Adolescence (with sample restricted to include only those with sleep data at the adolescent follow-up)

Adolescent Long Wake Episodes	Model 1		Model 2		Model 3	
	B	SE	B	SE	B	SE
Intercept	-1.36***	0.12	-1.40***	0.10	-1.39***	0.11
Long Wake Episodes T1	0.31***	0.05	0.30***	0.05	0.29***	0.05
Age	-0.10	0.11	-0.09	0.11	-0.09	0.11
Gender (Male)	0.19	0.15	0.24	0.14	0.17	0.15
Race(White)	-0.52**	0.16	-0.44**	0.16	-0.44**	0.14
Income to Needs			-0.11	0.09	-0.10	0.10
Parent Education			-0.09	0.08	-0.10	0.08
Neigh. Disadvantage	0.13*	0.05	0.10*	0.04	0.28***	0.07
Neigh. Disadvantage*Race					-0.35***	0.10

Note. Unstandardized estimates are shown. All continuous predictor variables are grand mean centered and z-scored to have a mean of zero and a standard deviation of 1 (n = 199). +p < 0.10. *p < 0.05. **p < 0.01. ***p < 0.001.

Table A9

Model Results for Sleep Duration in Adolescence (5 or more nights of data)

Adolescent Sleep Duration	Model 1		Model 2		Model 3	
	B	SE	B	SE	B	SE
Intercept	-38.99***	7.35	-36.58***	6.82	-25.00**	9.38
Sleep Duration (T1)	0.39***	0.11	0.39***	0.10	0.42**	0.13
Age	5.37⁺	2.98	4.96	3.00	5.23	3.14
Gender (Male =1)	-22.58**	8.61	-24.48**	8.67	-23.81**	9.10
Race (White =1)	22.19**	8.43	19.83*	8.59	1.58	8.87
Income to Needs			8.81	5.44	10.53	5.83
Parent Education			-7.37*	3.62	-7.47⁺	3.90
Neigh. Disadvantage	1.58	2.61	2.40	2.66	0.95	5.10
Neigh. Disadvantage*Race					0.56	7.99

Note. Unstandardized estimates are shown. All continuous predictor variables are grand mean centered and z-scored to have a mean of zero and a standard deviation of 1 (n = 339). +$p < 0.10$. *$p < 0.05$. **$p < 0.01$. ***$p < 0.001$.
